# Early results of a low-profile stent-graft for thoracic endovascular aortic repair

**DOI:** 10.1371/journal.pone.0240560

**Published:** 2020-11-19

**Authors:** Hazem El Beyrouti, Mario Lescan, Marco Doemland, Migdat Mustafi, Florian Jungmann, Tobias Jorg, Nancy Halloum, Bernhard Dorweiler

**Affiliations:** 1 Department of Cardiothoracic and Vascular Surgery, University Medical Center of the Johannes Gutenberg University Mainz, Mainz, Germany; 2 Department of Thoracic and Cardiovascular Surgery, University Medical Center Tübingen, Tübingen, Germany; 3 Department of Diagnostic and Interventional Radiology, University Medical Center of the Johannes Gutenberg University Mainz, Mainz, Germany; 4 Department of Vascular Surgery, University Medical Center, Cologne, Germany; Ospedale Sant'Antonio, ITALY

## Abstract

**Purpose:**

To assess outcomes of a low-profile thoracic stent-graft in the treatment of thoracic aortic pathologies.

**Methods:**

A retrospective analysis of all consecutive patients with aortic thoracic pathologies treated with the RelayPro device in two university hospitals between October 2018 and July 2019.

**Results:**

23 patients (65% men; mean age 63.4 ± 15 years) were treated. Pathologies included aortic dissections (n = 10), 5 residual type A (22%) and 5 type B (22%), 6 degenerative aortic aneurysms (26%), 4 penetrating aortic ulcers (17%), and aortic erosion, intramural hematoma and aortic rupture (n = 1 and 4% in each case). Two cases (9%) were emergent and two urgent. Proximal landing was achieved in zones 0 (4%), 1 (4%), 2 (43%), and 3 (26%). Five grafts were frozen elephant trunk extensions. Technical success was 100% with accurate device deployment in the intended landing zone of the aortic arch in all 23 patients and with no Ia/III endoleaks and three (13%) type II endoleaks. Apposition was adequate in 96%. Two patients had post-implantation syndromes (one fever, one leukocytosis). Mean follow-up was 11.6 ± 3.7 months (range, 2–16) with no other complications, secondary interventions or conversions to open surgery. There was no 30-day mortality and no aortic-related mortality; all-cause mortality was 4% during follow-up.

**Conclusion:**

A 3–4 French reduced profile in the current generation of stent-grafts facilitates TEVAR particularly in patients with smaller vessels access. Early safety and effectiveness outcomes are favorable, even in endpoints such as deployment accuracy and apposition which may be surrogates for longer-term clinical success and durability.

## Introduction

The advantages of thoracic endovascular aortic repair (TEVAR) with respect to open surgical repair include reduced procedure time, less blood loss, shorter hospital and intensive care stays, and earlier return to normal activities [1]. Its limitations as standard therapy, on the other hand, are not only the requirements for suitable pathology and aortic anatomy to ensure adequate landing zone, but also the complications of atherosclerosis and the size and tortuosity of access vessels. Small access arteries, calcification, tortuosity and prior vascular procedures increase the incidence of vascular injury including dissection, rupture, thrombosis, and intervention failure, and are associated with poor outcomes [2, 3]. Smaller access vessels are a predictor of perioperative complications and restrict eligibility for women, as well as Asian and young patients: populations who generally have more tortuous infrarenal aortas and smaller iliac diameters and who make up a great proportion of thoracic aortic pathologies [4]. Furthermore, smaller delivery sheaths in endovascular repair show less access vessel trauma and facilitate percutaneous access, which results is a significant reduction of the intervention times and less postoperative pain for the patient [5].

As TEVAR evolves, therefore, the current challenge is to improve not only the stent-graft but also the delivery system to deal with accurate and precise deployment, challenging aortic and access vessel anatomies, especially aortic or iliac tortuosity. The aim of this study was to report early outcomes of a new low-profile thoracic stent-graft.

## Materials and methods

### Study design

This was a retrospective, non-randomized, dual center, observational, single-arm study of all consecutive patients with thoracic aortic pathologies treated with a single low-profile endograft in two university hospitals between October 2018 and July 2019. The aim was to evaluate technical success: successful stent-graft delivery and deployment accuracy at the intended landing zone with stent-graft apposition and without unintentional vessel coverage, type Ia or III endoleaks, maldeployment and access vessel complications [6]. Accurate device deployment was defined as deployment ≤ 5 mm from the target anatomical position along the greater curvature, and stent-graft apposition with the absence of a bird beak sign, which is defined as a gap > 5 mm between the inner aortic curvature and the endograft at the first postoperative CT scan [7, 8].

Other safety and effectiveness endpoints included the rate of secondary procedures and major adverse events such as: myocardial infarction, stroke, paraplegia, aortic rupture, retrograde dissection, resuscitation, ventilation more than 72 hours or reintubation, tracheostomy, renal failure (permanent dialysis), organ failure, access or wound complications requiring secondary intervention were assessed. Other performance endpoints included the incidence of complications such as stent graft-migration, and failure of device integrity, stent thrombosis, obstruction, twisting, compression, erosion, graft infection, aortic rupture, endovascular or open surgical secondary interventions due to endoleak, all-cause and aortic-related mortality during the follow-up.

Approval from institutional ethics committee was obtained in both centers: from the responsible ethics committee of the State Medical Association of Rheinland Pfalz was obtained for data analysis (2020–14946), Mainz, Germany; and from the Ethics Committee at the Medical Faculty of the Eberhard-Karls-University and at the University Hospital Tübingen (347/2018BO), Tübingen, Germany. This was a retrospective study of medical records, data were anonymized before analysis and the requirement for informed consent specific to this study was waived.

### Device description and procedure

The Relay stent-graft (Terumo Aortic, Sunrise, Fla, USA) has been available since 2009 in Europe and 2012 in the USA to treat aortic pathologies and the early and midterm outcomes of the first and the second generation devices (RelayPlus) were encouraging despite high profile introducer sheaths (22—26F) [9]. RelayPro is the third generation device with a 3—4F reduction in outer diameter (now 19—23F) and a shorter S-Bar to accommodate curved aortic anatomies [10]. As in previous generations, the proximal end configuration can be used with bare stents (for alignment, not fixation as it has low radial load) or without (non-bare stent, NBS). Both configurations are available in diameters between 22 and 46 mm and lengths between 100 and 250 mm. A distinguishing element of the Relay device is its deployment through a dual-sheath system: one stable outer sheath for support during advancement and a second inner, flexible sheath which allows atraumatic navigation to and across the aortic arch [11].

Vascular access was through one or both sides with direct surgical femoral exposure. Spinal cord protection in elective procedures comprised preoperative cerebrospinal fluid (CSF) drainage (intracranial pressure ≤10 mmHg) in cases where aorta coverage exceeded 20 mm, the LSA was covered, there was prior infrarenal repair or internal arteries occlusion; and postoperative maintenance of mean arterial blood pressure ≥75–80 mmHg for all patients [12–14].

In emergency cases, we also maintained mean arterial blood pressure ≥75–80 mmHg; CSF was drained only if spinal ischemia occurred postoperatively. All patients received 5000 units of heparin.

### Image analysis

Diagnosis was confirmed by multislice high-resolution spiral computed tomography angiography (CTA). Multiplanar and three-dimensional workstation reconstructions were used to evaluate aortic pathology. Standard of care CTA is baseline for case planning, before discharge, 3 months, 6 months and yearly thereafter. In Mainz, images are evaluated with Sectra Workstation IDS 7 (Sectra AB, Linkoeping, Sweden) with automatic generation of centerline of flow (CLF); in Tübingen, with Osirix MD software (Pixmeo, Bernex, Switzerland). Case planning included access vessel anatomy evaluation including diameter (lowest diameter between the femoral access artery and the aortic bifurcation on the access site); calcification (significant if vessel diameter area shows a stenosis > 50%); and tortuosity index of both thoracic aorta and iliac arteries (ratios between CLF distance [thoracic aorta: from the fourth thoracic vertebrae to the offspring of the celiac trunk; iliac arteries: from the aortic bifurcation to the offspring of inferior epigastric arteries) and the shortest distance between the same measurements). Tortuosity index values were interpreted as mild (<1.1), moderate (1.11–1.18) or severe (≥1.19). Aortic arch classification was performed in respect to STORAGE guidelines and the Modified Arch Landing Areas Nomenclature (MALAN) classification based on hemodynamic features [15].

### Statistical analysis

Statistical computations and figures were done using GraphPad prism version 7.0a for Mac (GraphPad Software, La Jolla, CA, USA), wizard pro data analysis version 1.9.7 (Evan Miller, Chicago, IL) and SPSS 22.0 for MAC (SPSS, Chicago, IL, USA). All frequency data are presented as percentages and all continuous data as the mean ± standard deviation: data were tested for normality and presented according to the distribution. The confidence interval is 95%.

## Results

### Demographic parameters and comorbidities

Table 1 summarizes patient demographics, comorbidities, preoperative status, and anatomical characteristics. The mean age of the cohort was 63.4 ± 15 years. Eight patients from the cohort were female (35%) and 17 patients (74%) were American Society of Anesthesiologists (ASA) class III/IV with the comorbidities including arterial hypertension (n = 21; 91%), nicotine abuse (n = 6; 26%), and hyperlipidemia (n = 10; 44%). Two patients had a history of cerebrovascular events without residual disability at the time of intervention; one had a history of transient ischemia attack (n = 3, 13% with prior neurological injury).

**Table 1:** Patient demographics, medical history, and aortic characteristics.

	N = 23
Male	15 (65%)
Age (years)	63.4 ± 15
Body mass index (kg/m^2^)	36.3 ± 43.4
Hypertension	21 (91%)
Hyperlipidemia	10 (43%)
Nicotine abuse	6 (26%)
Chronic renal insufficiency	4 (17%)
COPD	3 (13%)
Coronary artery disease	3 (13%)
Diabetes mellitus	3 (13%)
Neurological injury	3 (13%)
Myocardial infarction	2 (9%)
Dialysis	1 (4%)
Peripheral arterial disease	1 (4%)
**ASA class**	
II	6 (26%)
III	10 (43%)
IV	7 (30%)
**Aortic pathology characteristics**	
Degenerative aneurysm	6 (26%)
Residual Type A dissection	5 (22%)
Type B dissection	5 (22%)
Penetrating aortic ulcer	4 (17%)
Aortic erosion	1 (4%)
Intramural hematoma	1 (4%)
Aortic rupture	1 (4%)
**Aortic arch classification**	
Type I	4 (17%)
Type II	9 (39%)
Type III	5 (22%)
Prosthetic graft	5 (22%)
Access vessel diameter (mm)	7.7 ± 1.3 (5–10)
Iliac tortuosity	1.35 ± 0.23 (1.05–2.04)
Aortic tortuosity	1.19 ± 0.77 (1.09–1.37)
Aortic calcification > 50%	5 (22%)
**Prior surgery**	
Frozen elephant trunk	5 (22%)
Ascending aorta replacement	9 (39%)
Infrarenal aortic replacement	1 (4%)
EVAR	1 (4%)
Endovascular thoracoabdominal repair	1 (4%)
TEVAR	1 (4%)

Values are n (%) or mean ± standard deviation and (range).

ASA, American Society of Anesthesiologists; COPD, Chronic Obstructive Pulmonary Disease; EVAR, (infrarenal) endovascular aortic repair; SD, standard deviation; TEVAR, thoracic endovascular aortic repair.

The patients were treated for residual type A (TAAD; n = 5, 22%) and type B aortic dissections (TBAD; n = 5, 22%) degenerative aortic aneurysms (n = 6, 26%), penetrating aortic ulcers (n = 4, 17%), and aortic wall erosion caused by previous TEVAR, intramural hematoma and aortic rupture (n = 1 or 4% in each case). Two cases (9%) were emergent (one aortic erosion, one aortic rupture), two case were urgent (9%) (one symptomatic TBAD, one IMH; two cases (9%) were reinterventions for endoleaks after previous endovascular repair. Five procedures (22%) were reinterventions for late distal stent induced new entries or distal true lumen collapse after frozen elephant trunk (FET). Two patients (9%) had prior infrarenal aortic repair.

### Operative results

The procedure was performed under general anesthesia in 17 (74%) patients and analgosedation in 6 (26%). Mean operative time was 83 ± 41 min (excluding concomitant bypass/debranching procedures) and 259 ± 51 min (including concomitant bypass/debranching); mean fluoroscopy time was 16 ± 10 min, mean contrast used was 106 ± 53 ml. Eight patients (35%) had access vessel diameter ≤7 mm. The mean tortuosity index of the cohort was severe (1.19 ± 0.77) and 22% (n = 5) had significant calcification. An abdominal octopus bypass to reroute the visceral and the renal vessels from the common right iliac artery was performed in one patient as part of an extent I TAAA repair. Spinal cord protection measures included: CSF drainage and induced hypertension (n = 11, 48%; one Z0, six Z2 and three Z3) (S1 Video) and pharmacologically-induced hypertension for all other patients.

An average of 1.35 stent-grafts were implanted to cover a mean thoracic aorta length of 207.8 ± 96 mm (range, 97–550); five patients (22%) needed a second device for complete coverage; 13/28 (46%) devices were tapered, and most were NBS (n = 20; 89%). Deployment was under rapid ventricular pacing in most cases (n = 14, 70%; one Z1 and Z2 each, ten Z2 and two Z3).

**Fig 1:**
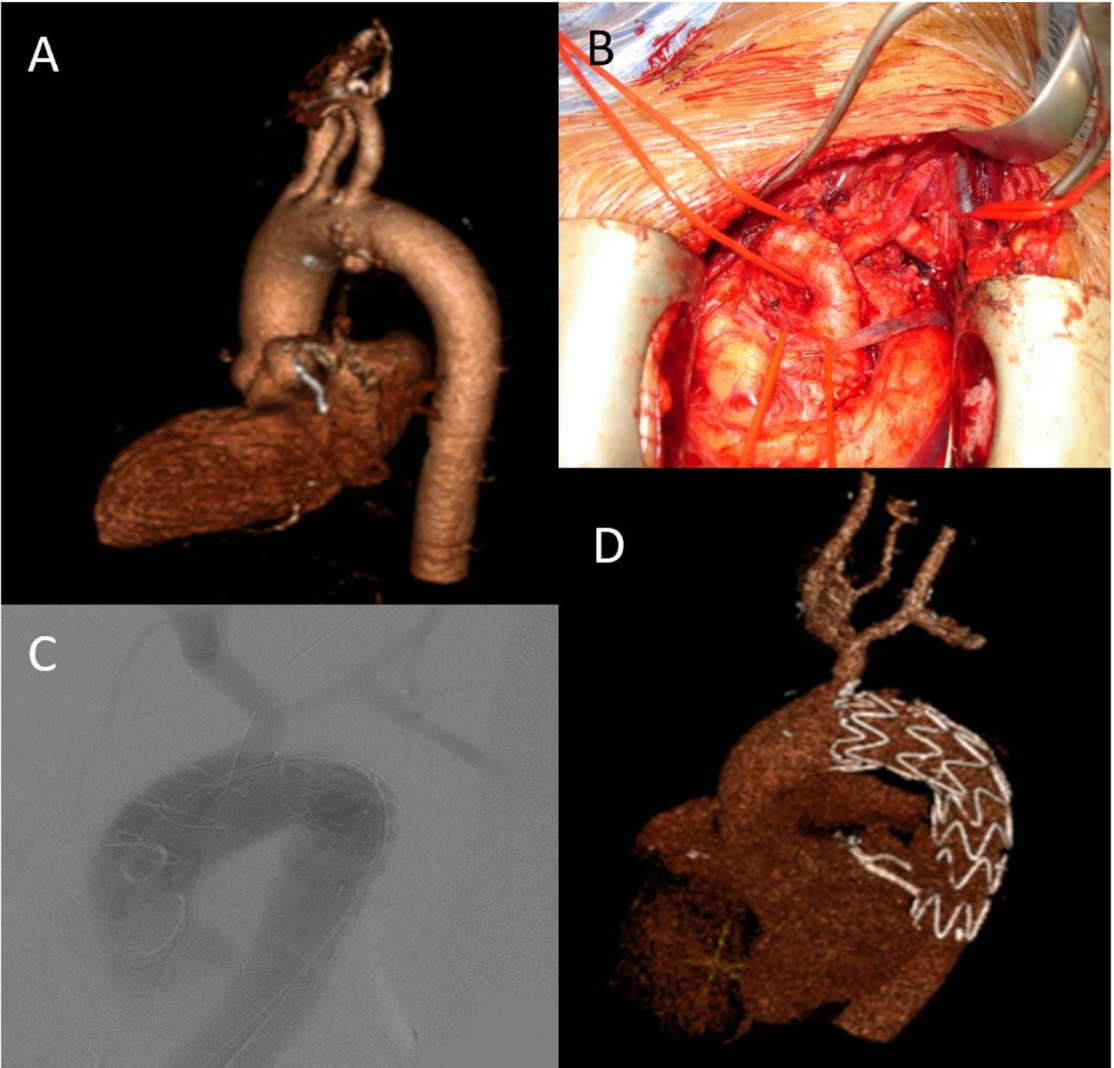
Preoperative, intraoperative and postoperative imaging. A- Preoperative volume-rending 3D computed tomography reconstruction of aorta showing penetrating atherosclerotic ulcer. B- Intraoperative, native double debranching of left subclavian artery and left carotid artery to the brachiocephalic trunk via mini-sternotomy. C- Intraoperative, Digital subtraction angiography (DSA) RelayPro in zone 1. D- Postoperative, volume-rending 3D-CT reconstruction of aorta of RelayPro in zone 1.

Table 2 details proximal landing zones according to the MALAN classification. The left subclavian artery (LSA) was intentionally covered in 12 patients (52%); 10/12 (83%) were revascularized intraoperatively, one had left common carotid artery (LCCA) and LSA direct debranching and one had total debranching via mini-sternotomy (Fig 1). Five patients (22%) had concomitant LSA occlusion. Post-implantation ballooning was limited to three patients (13%). Table 3 gives operative details for all patients.

**Table 2:** TEVAR landing zones and aortic arch configurations in 23 patients.

Ishimaru zone	Arch type				
	Type I	Type II	Type III	post-FET	
				FET zone 2	FET zone 3
**Zone 0**	1	0	0	-	-
**Zone 1**	1	0	0	-	-
**Zone 2**	1	4	5	4	-
**Zone 3**	1	5	0	-	1

MALAN classification with post-FET considerations: FET zone 2 is distal to the LCCA after extra-anatomic LSA bypass and FET zone 3 is at T4, distal to the LSA.

FET, frozen elephant trunk; LCCA, left common carotid artery; LSA, left subclavian artery; MALAN, Modified Arch Landing Areas Nomenclature.

**Table 3:** Procedural details.

	N = 23	
Elective	19 (83%)	
Emergent	2 (9%)	
	Urgent 2 (9%)	
General anesthesia	17 (64%)	
Analgosedation	6 (26%)	
Ventricular rapid pacing	14 (70%)	
Prophylactic CSF drainage	11 (48%)	
**Procedure times (min)**		
Overall (n = 23)	167.4 ± 100	31–372
Without concomitant procedures (n = 11)	83 ± 41	31–150
With concomitant procedures (n = 12)	259 ± 51	189–372
**Radiographic details**		
Dose area product (μGym^2^)	2209 ± 1430	456–5597
Fluoroscopy time (min)	16 ± 10	5–43
Contrast used (mL)	106 ± 53	35–218
Complications		
Blood loss > 1L	1 (4%)	
Intensive care	3 (13%)	
Hospital stay (days)	12.1 ± 6.1	5–30
**Device**		
RelayPro NBS	23/28 (82%)	
RelayPro (bare stent)	5/28 (18%	
RelayPro (tapered)	13/28 (46%)	
Technical success	23 (100%)	
Bird beak (> 5 mm)	1 (4%)	
Deployment ≤ 5 mm from the target	23 (100%)	

Values are n (%) or mean ± standard deviation and range.

CSF, cerebrospinal fluid.

### In-hospital results

Technical success was achieved in 100% with successful stent-graft delivery and lesion exclusion without type Ia or III endoleak in all patients. Accurate device deployment (≤ 5 mm from the target position) was achieved in all patients (100%). Stent-graft apposition in the aortic arch was 96%; there was one case of bird beak measuring 6 mm (in the absence of type Ia endoleak) in a patient with a II/2 MALAN classification; 15 patients had 100% apposition, seven had non-significant bird-beak gap with a mean of 3.5 ± 1.3 mm. There were three (13%) type II endoleaks and no endoleaks type Ia or III.

No other unplanned adjunctive procedures were necessary: no vascular access complication, retrograde dissections or false lumen and aneurysm ruptures. Two patients (9%) experienced post-implantation syndrome: one with fever until postoperative day (POD) 4 with no sign of infection in blood tests: another with observed leukocytosis. After antibiotic therapy, both patients were discharged without fever.

Three (13%) patients were admitted to the intensive care unit (ICU) and they stayed for a mean of 3.5 ± 1.8 (median 2) days; two required ventilation for a mean 28 ± 25.4 (median 28) hours. One TAAA patient (with preoperative respiratory failure and history of dialysis due to chronic renal insufficiency) required prolonged ventilation (46 hours) and renal support and spent nine days in the ICU. He was discharged to a rehabilitation center on POD 30. Intraoperative complications were limited to significant blood loss in the patient with aortic erosion, who required 2750 mL of blood product transfusion; in total, three patients required transfusion with a mean of 3.8 ± 3 (median 2.5) units of 250 mL. All other patients were monitored and observed postoperatively in the surgical ward for a mean of 12.1 ± 6.1 (median 10) days until discharge.

### Follow-up results

As shown in Table 4, mean follow-up was 11.6 ± 3.7 months (range, 2–16 months) with no other secondary interventions or conversions to open surgery and no new endoleaks: the three type II endoleaks were not treated and persisted under observation. No aneurysm enlargement was observed to date. Thirty-day mortality was not observed. Freedom from all-cause mortality was 96% and freedom from aortic-related mortality was 100%. One non-aortic-related death occurred at 2.5 months. There was no migration ≥5 mm; 12 patients had 0 mm migration, a mean 2.4 ± 1.5 mm was seen in 11 patients. Device related complications including stent fractures, endograft thrombosis, obstruction, compression and graft infection were not observed in our cohort. There were no aortic ruptures, endovascular reinterventions or conversion to open repair during the mid-term follow-up period. Retrograde dissection, paraplegia, ruptures, myocardial infarction, paraplegia or paralysis were also not seen.

**Table 4:** Outcomes.

	N = 23
Follow-up (months)	11.6 ± 3.7 (2–16)
30-day mortality	0
One-year all-cause mortality	1 (4%)
One-year aortic-related mortality	0
**Endoleaks**	
Type Ia	0
Type II	3 (13%)
Type III	0
**Complications**	
Post-implantation syndrome	2 (9%)
Spinal cord ischemia	0
Cerebrovascular	0
Myocardial infarction	0
Retrograde dissection	0
dSINE	0
Thrombosis, occlusion, etc	0
Rupture	0
Renal failure	0
**Other parameters**	
Device integrity	23 (100%)
Secondary interventions	0
Migration (>5 mm)	0

Values are n (%) or mean ± standard deviation and (range).

dSINE, distal stent-graft induced new entry; TEVAR, thoracic endovascular aortic repair.

## Discussion

A new generation of thoracic stent-grafts is overcoming some of the limitations of TEVAR noted to date. Expectations of these devices are also increasing and new and more precisely-measured parameters such as alignment—which we understand as both deployment accuracy and apposition—as well as new complications (such as dSINE) have joined established endpoints such as technical success and endoleaks because they may flag potential failure before clinical manifestation. It is generally accepted that stent-graft migration less than the original standard of 10 mm may be relevant; reporting exact measurements contributes to the establishment of a new reporting standard with clinical relevance [16].

In parallel to device improvement, physician expertise has increased to include innovative strategies for alternative access for graft deployment such as the cardiac apex or the ascending aorta [17]. Along with increasingly complex procedures going further into the aortic arch, combining open and endovascular surgery, these changes have made it increasingly difficult to compare results with earlier TEVAR reports. In addition, the longer-term results and outcomes of these innovations remain to be demonstrated. Consequently, observational, real-world experience has some value in this respect [18].

Much progress to lower profile stent-grafts has already been made and the Cook Medical Zenith Alpha and Medtronic Valiant Navion (both 18–22/23F outer diameter) have demonstrated encouraging early and midterm results [19, 20]. In a prospective study of 110 patients (42% women, 38% Asian) treated with the Zenith Alpha, mean access vessel diameter was 6.7 ±1.6 mm and 36% of implants were percutaneous [21]. Almost 90% of implants were percutaneous in a later study of 70 patients treated with the Zenith Alpha and 22.3 ± 15.9 months follow-up: 6% required balloon dilation of the iliac arteries, 4% required iliofemoral conduit: 4% had access vessel complications [19]. In 87 patients (40% female) treated with the Valiant Navion, 51% were accessed percutaneously, four patients required iliac stenting and there was no access or deployment failure [20]. In a European multicenter registry of 100 patients treated with a Gore cTAG (18—24F), iliac conduits were needed in 5% of patients [22].

There were no adjunctive techniques in this series despite a mean access vessel diameter of 7.7 ± 1.32 mm (range 0.5–1); compared with 9.1 (6–13) mm previously reported with RelayPro [10]. Most patients had severe iliac and aortic tortuosity but there was no vessel-access related complication in this series, confirming a generally low incidence with all the low-profile devices reported so far: 3% [23], 5.7% [20], 6% [10] and 7% [22].

We used 13 (46%) tapered stent-grafts and a mean 1.35 devices (mostly NBS) to cover a mean aortic length of 207.8 mm; the availability of Relay devices as long as 250 mm reduces overlapping and consequently the risk of type III endoleak. This low mean number of units per procedure contributes to lower total TEVAR costs and is common across the studies using Relay.

TEVAR outcomes are determined in part by stent-graft deployment accuracy and apposition. As experience with TEVAR grows, so too does the sophistication with which we measure outcomes as well sensitivity to variability in pathology, anatomy and use; the Gore cTAG with an active control deployment system demonstrated 100% accuracy in 20 patients with landing zone ≤3 and aortic arch types I (n = 1), II (n = 10), and III (n = 9) [24]. The previous generation RelayPlus NBS demonstrated accurate deployment in the aortic arch in 78 patients with a wide variety of thoracic pathologies involving arch zones 2 (44%) and 3 (54%): aortic arch type was not specified but the accurate deployment ≤5 mm from the target vessel was found in 82% patients and there was no type Ia endoleak despite bird beak (5 mm or more) in 4% of the cohort immediately after implant and 11% of the patients during the follow-up [7]. Precise measurement of alignment is another important step forward in TEVAR outcomes reporting and understanding better device performance in terms of clinical relevance; one report gave a 50% risk of endoleak at a bird beak length of 9.5 mm and an 80% risk at 14 mm, while more recently a 5-mm minimum was defined as substantial [8, 25]. In our cohort, the RelayPro delivery system (which allows for small incremental movements) was used in ‘suboptimal’ landing areas (according to the MALAN classification) but with 100% deployment accuracy (all ≤5 mm from the intended position), no type Ia endoleak, and 96% stent-graft apposition.

These results are favorable and/or comparable to what has been reported so far with other latest-generation or low-profile devices. There was no 30-day mortality as compared to 2.3%, 9% and 10% reported by other authors [20, 22, 23]. We saw no aortic rupture or stent fractures, again in line with the low incidences reported to date (1% and 1.1% for rupture and 0.9% for fracture) [20–22]. Freedom from aortic-related mortality and the reintervention were 100%, and freedom from all-cause mortality was 93% similar to 80–95% reported previously [21, 22]. Finally, retrograde dissection is a devastating complication of TEVAR and the incidence is low overall, but more common as total TEVAR numbers increase. Like Mariani et al., we observed no retrograde dissection [24]. In comparison, other TEVAR studies have reported incidences of 1–2.3% [7, 20, 22].

This initial experience of the new low-profile stent-graft adds to the literature on the latest-generation devices. The study is limited by its nature as retrospective, observational, and with a small series of patients without long-term follow-up. Within these limitations, these early, real-world results contribute to the literature in two regards: updating references for the remaining perioperative concerns about access; and including more detailed results on issues of deployment and apposition at a time of greater scrutiny and emergence of more and more succinct endpoints.

## Conclusion

RelayPro showed good mid-term performance in the treatment of a variety of thoracic pathologies by overcoming the challenge of difficult access anatomies, which is regarded as one of the limitations of TEVAR. Accurate deployment with favorable apposition even in hostile aortic arches contributed to low rates of early and mid-term complications.

## Supporting information

S1 Video Implantation of a RelayPro in zone 0. (MP4)
